# Atlantic City in 1901

**Published:** 1900-11

**Authors:** 


### ATLANTIC CITY IN 1901.

THE JOURNAL is glad to announce, just as it goes to press with this number, that Atlantic City, N. J., will be the place of meeting in 1901. Sufficient of the votes of the Executive Committee have been polled to decide in that city's favor, and there will be a general rejoicing all along the eastern coast at this decision. There will be a generous welcome in that Mecca by the sea to every veterinarian who attends, and there will be a wide-open hospitality on the part of the profession, the foremost citizens of that city, and the cheapest railroad rates of any year in Association history.

